# Novel diagnostic value of circulating *miR-18a* in plasma of patients with pancreatic cancer

**DOI:** 10.1038/bjc.2011.453

**Published:** 2011-11-01

**Authors:** R Morimura, S Komatsu, D Ichikawa, H Takeshita, M Tsujiura, H Nagata, H Konishi, A Shiozaki, H Ikoma, K Okamoto, T Ochiai, H Taniguchi, E Otsuji

**Affiliations:** 1 Division of Digestive Surgery, Department of Surgery, Kyoto Prefectural University of Medicine, 465 Kajii-cho, Kawaramachihirokoji, Kamigyo-ku, Kyoto 602-8566, Japan; 2 Department of Surgery, Kyoto Second Red Cross Hospital, 355-5 Kamanzadoori Marutacho Hruobicho, Kamigyo-ku, Kyoto, Japan

**Keywords:** pancreatic cancer, microRNA, plasma, biomarker

## Abstract

**Background:**

Several recent studies have demonstrated that microRNAs (miRNAs) are stably detectable in the plasma/serum. We hypothesised that miR-18a in the plasma is a potential biomarker in patients with pancreatic cancer.

**Methods:**

miR-18a is located in the miR-17–92 cluster and reported to be highly expressed in pancreatic cancer tissues. This study was divided into three parts: (1) Confirmation of higher miR-18a levels in primary pancreatic cancer tissues and cell lines than in normal pancreatic tissues and a human fibroblast cell line. (2) Evaluation of the plasma miR-18a assay using quantitative RT–PCR by comparing plasma results obtained from 36 patients with pancreatic cancer and from 30 healthy volunteers. (3) Evaluation of the assay for monitoring tumour dynamics in patients with pancreatic cancer.

**Results:**

(1) The expression of miR-18a was significantly higher in pancreatic cancer tissues (*P*=0.012) and pancreatic cancer cell lines (*P*=0.015) than in normal tissues and fibroblasts. (2) Plasma concentrations of miR-18a were significantly higher in pancreatic cancer patients than in controls (*P*<0.0001). The value of the area under the receiver-operating characteristic curve (AUC) was 0.9369. (3) Plasma levels of miR-18a were significantly lower in postoperative samples than in preoperative samples (*P*=0.0077).

**Conclusion:**

Circulating miR-18a might provide new complementary tumour markers for pancreatic cancer.

Pancreatic cancer is the fifth leading cause of cancer deaths in Japan and the fourth leading cause of cancer-related deaths in the United States (Jemal *et al*, 2003; Hirata *et al*, 2007). Recent improvements of surgical techniques and perioperative management have reduced operation-related deaths during the perioperative periods; however, pancreatic cancer has extremely poor prognosis because it develops local invasiveness and metastases to distant organs in the early stage of clinical course. Therefore, primary tumours must be detected at an early stage, and recurrent disease must be diagnosed when it is still minimal or clinically occult, to improve the survival rates for patients with pancreatic cancer.

Recent gene expression studies have identified a small number of genes that are differentially expressed in pancreatic cancer (Almoguera *et al*, 1988; Thayer *et al*, 2003; Jones *et al*, 2008). However, in a clinical setting, few molecules have been assayed as therapeutic and/or diagnostic biomarkers. Conventional serum tumour markers, such as carcinoembryonic antigen (CEA) and carbohydrate antigen 19-9 (CA19-9), have been used for diagnostic assays (Satake *et al*, 1985) in the early detection and monitoring of pancreatic cancer. However, these serum tumour markers lack sufficient sensitivity and specificity to facilitate the early detection of cancer. Therefore, the significance of detecting novel biomarkers using a less invasive diagnostic assay for pancreatic cancer should be emphasised.

In recent years, several studies have shown that microRNAs (miRNAs), which are involved in tumourigenesis and in the development of various cancers, are detectable in the plasma/serum (Calin and Croce, 2006; Chen *et al*, 2008; Filipowicz *et al*, 2008). Mitchell *et al* (2008) clearly demonstrated that circulating miRNAs originate from cancerous tissues, and are protected from endogenous RNase activity by unknown mechanisms. Indeed, many studies have demonstrated the presence of circulating miRNAs and their potential use as novel biomarkers of cancers, such as prostate cancer (Mitchell *et al*, 2008), leukaemia (Lawrie *et al*, 2008), oral cancer (Wong *et al*, 2008), pancreatic cancer (Wang *et al*, 2009), colorectal cancer (Ng *et al*, 2009), ovarian cancer (Resnick *et al*, 2009), lung cancer (Hu *et al*, 2010), breast cancer (Heneghan *et al*, 2010), gastric cancer (Tsujiura *et al*, 2010) and oesophageal cancer (Komatsu *et al*, 2011). These findings should open up a new and interesting field in the screening and monitoring of pancreatic cancer patients. However, to date, there have been few reports on the role of circulating miRNAs in the plasma of patients with pancreatic cancer.

In this study, we investigated the miR-17–92 cluster. This cluster of miRNAs was reported to have potential oncogenetic function in various tumours, with its elevation either being caused by genome amplification or by transcriptional activation by MYC (Woods *et al*, 2007); therefore, we hypothesised that plasma miRNAs, which are located in the miR-17–92 cluster, might be a potentially useful biomarker in patients with pancreatic cancer.

The miR-17–92 cluster consists of seven miRNAs, namely miR-17-5p, miR-17-3p, miR-18a, miR-19a, miR-19b, miR-20a and miR-92a. In selecting a putative oncogenic miRNA of the miR-17–92 cluster, as a novel candidate target for this plasma miRNA assay, we excluded three miRNAs, namely miR-17-5p, miR-20a and miR-92a, because these have already been reported as plasma biomarkers (Heegaard *et al*, 2011; Moussay *et al*, 2011; Ohyashiki *et al*, 2011). Moreover, we reviewed previous reports regarding miRNAs associated with the pancreatic cancer cell line and tissues. Of the remaining four, namely miR-17-3p, miR-18a, miR-19a and miR-19b, only miR-18a is highly expressed in pancreatic cancer cell lines and tissues (Szafranska *et al*, 2007); therefore, we confirmed miR-18a as a candidate for this plasma assay.

The miR-18a has been found to be significantly upregulated in gastric cancer (Yao *et al*, 2009), diffuse large B-cell lymphoma (Alencar *et al*, 2011), urothelial carcinomas of the bladder (Ayala de la Pena *et al*, 2011), nasopharyngeal carcinoma (Li *et al*, 2011), hepatocellular carcinoma, pancreatic carcinoma and colorectal carcinomas (Motoyama *et al*, 2009). In colorectal cancer tissues, patients with overexpressed miR-18a had a poorer clinical prognosis. Moreover, miR-18a was confirmed to directly target ER*α* and showed higher levels of expression in ER*α*-negative clinical tumours (Leivonen *et al*, 2009). Interestingly, miR-18a had a pro-proliferation effect on hepatocellular carcinoma cells, but an inhibitory effect on breast cancer cells (Leivonen *et al*, 2009).

In this study, we investigated whether the concentration of circulating miRNAs in plasma samples could be used to screen for cancer by comparing findings in pancreatic cancer patients and volunteer controls and by monitoring tumour dynamics. Consequently, we clearly demonstrate its potential usefulness. Our results provided evidence that the plasma level of miR-18a could be used to distinguish pancreatic cancer patients from healthy individuals with a clinically satisfactory degree of sensitivity and specificity.

## Materials and methods

### Patients and samples

This study was approved by the Institutional Review Board of the Kyoto Prefectural University of Medicine, and each subject provided signed informed consent. Between January 2010 and June 2011, 36 plasma samples of pancreatic cancer patients and 30 control samples were collected at the Kyoto Prefectural University of Medicine and the Kyoto Second Red Cross Hospital. The patients' characteristics with respect to age, sex, histopathology and stages of disease are described in Table 1. Pre-operative plasma samples were collected from 27 patients with pancreatic cancer, including 21 who underwent a pancreatectomy and 6 patients who received palliative surgery, and 9 non-operative patients who had advanced stage and recurrent disease. In all, 11 patients underwent chemotherapy before collecting plasma samples. No patients underwent chemoradiotherapy. All patients were pathologically diagnosed as having pancreatic cancer using surgical specimens and biopsies. Two patients were diagnosed with adenosquamous carcinoma, and one patient was diagnosed with anaplastic carcinoma. The remaining 33 patients were diagnosed with adenocarcinoma.

From patients who underwent surgery, 19 pancreatic cancer specimens were collected. We also collected five normal tissue specimens from an adjacent benign pancreatic tumour, such as insulinoma, which was resected. As a control, plasma was collected from 30 volunteers. Volunteers were medical personnel and patients with a benign disease such as cholelithiasis. They underwent medical examinations and did not have any pancreatic disease or other cancerous disease. The stage of tumours was assessed according to the Union Internationale Contre le Cancer classification (Sobin *et al*, 2009).

### Collection of heparin-treated blood plasma and pancreatic tissue

Blood was collected from patients and controls in sodium heparin tubes (BD Vacutainer, Franklin Lakes, NJ, USA) and immediately subjected to a three-spin protocol (1500 r.p.m. for 30 min, 3000 r.p.m. for 5 min and 4500 r.p.m. for 5 min) to prevent contamination by cellular nucleic acids. Plasma samples were then stored at −80°C until further processing. All resected specimens were fixed in formalin and embedded in paraffin (FFPE) for pathological diagnosis. Tissues adjacent to specimens were evaluated histologically according to the criteria of the World Health Organization. In all cases, two pathologists were in agreement with regard to pathological features and both confirmed the diagnoses.

### RNA extraction

Total RNA was extracted from cultured cells and 400 *μ*l of plasma using a mirVana PARIS kit (Ambion, Austin, TX, USA), and eluted into 100 *μ*l of pre-heated (95°C) elution solution according to the manufacturer's directions. For FFPE tissues, total RNA was extracted from 4 slices of 15-*μ*m thickness (total thickness of 60 *μ*m) using a RecoverAll Total nucleic acid isolation kit (Ambion), and finally eluted into 60 *μ*l of elution solution according to the manufacturer's instructions.

### Protocol for detection of miRNAs

The amounts of miRNAs were quantified in duplicate through qRT–PCR using the human TaqMan MicroRNA assay kits (Applied Biosystems, Foster City, CA, USA). The reverse-transcription reaction was carried out using a TaqMan MicroRNA reverse-transcription kit (Applied Biosystems) in 15 *μ*l containing 5 *μ*l RNA extract, 0.15 *μ*l 100 mM dNTPs, 1 *μ*l Multiscribe reverse transcriptase (50 U *μ*l^−1^), 1.5 *μ*l 10 × reverse transcription buffer, 0.19 *μ*l RNase inhibitor (20 U *μ*l^−1^), 3 *μ*l gene-specific primer and 4.16 *μ*l nuclease-free water. For synthesis of cDNA, reaction mixtures were incubated at 16°C for 30 min, at 42°C for 30 min and at 85°C for 5 min and then held at 4°C. Next, 1.33 *μ*l cDNA solution was amplified using 10 *μ*l of TaqMan 2 × Universal PCR Master Mix with no AmpErase UNG (Applied Biosystems), 1 *μ*l of gene-specific primers/probe and 7.67 *μ*l of nuclease-free water in a final volume of 20 *μ*l. Quantitative PCR was run on a 7300 real-time PCR system (Applied Biosystems) and the reaction mixtures were incubated at 95°C for 10 min, followed by 40 cycles of 95°C for 15 s and 60°C for 1 min. The cycle threshold (Ct) values were calculated using the SDS 1.4 software (Applied Biosystems).

The amounts of miRNAs in the plasma were calculated on a standard curve constructed using synthetic miRNAs, mirVana miRNA reference panel (Ambion). Standard reference miRNAs were amplified for each reaction. However, the expression of miRNAs from tissue samples was normalised using the 
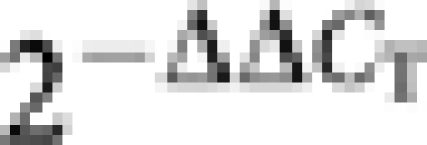
 method relative to U6 small nuclear RNA (RNU6B). ΔCt was calculated by subtracting the Ct values of RNU6B from the Ct values of the miRNAs of interest. ΔΔCt was then calculated by subtracting the ΔCt of the normal pancreatic tissue from the ΔCt of pancreatic cancer tissues. The change in gene expression was calculated with the equation (2) 
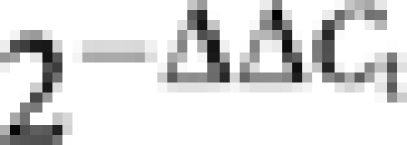
 (Livak and Schmittgen, 2001; Pfaffl, 2001).

### Pancreatic cancer cell lines and culture

The pancreatic cancer cell lines NOR-P1, PK-45H, KP4-1, PANC-1 and PK-59 and the fibroblast cell line WI-38 were purchased from the RIKEN Cell Bank (Tsukuba, Japan). NOR-P1and WI-38 cells were cultured in Dulbecco's minimum essential medium: F12 medium and the others in Roswell Park Memorial Institute (RPMI)-1640 medium (Sigma, St Louis, MO, USA). All media were purchased from Sigma, and supplemented with 100 ml l^−1^ FBS (Trace Scientific, Melbourne, Victoria, Australia). All cell lines were cultured in 50 ml l^−1^ carbon dioxide at 37°C in a humidified chamber.

### CA19-9 assay

In all, 2ml of blood was drawn from each pancreatic cancer patient, and the serum component extracted. Carbohydrate antigen 19-9 was quantified by the Kyoto Prefectural University of Medicine Clinical Laboratory using the ADVIA Centaur 19-9 assay (Siemens Diagnostic Healthcare, Deerfield, IL, USA). The standard range of CA19-9 concentrations in our institution was 0.0–37.0 ng ml^−1^.

### Statistical analysis

The Mann–Whitney *U*-test and Kruskal–Wallis *H*-test for unpaired data were performed to compare plasma miRNA concentration and miRNA ratio. The Wilcoxon test was used to compare the paired plasma samples before and 1 month after pancreatectomy. A *P*-value of 0.05 was considered significant. Receiver operating characteristic (ROC) curves and the area under the ROC curve (AUC) were used to assess the feasibility of using plasma miRNA concentrations as diagnostic tools for detecting pancreatic cancer.

## Results

### Study design to develop a novel biomarker of plasma miRNA

**Figure 1:**
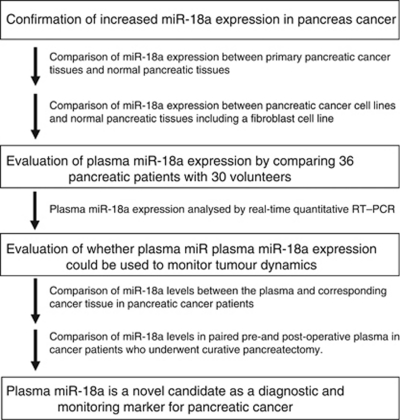
Study design to develop a novel biomarker of the plasma microRNA.

The study design is summarised in Figure 1. This study was divided into three parts: (1) Confirmation of the higher miR-18a levels in primary pancreatic cancer tissues and cell lines than in normal pancreatic tissues and human fibroblasts. We compared miR-18a levels between primary pancreatic cancer tissues and normal pancreatic tissues. In addition, we compared miR-18a levels between pancreatic cancer cell lines and a fibroblast cell line. (2) Evaluation of the plasma miR-18a assay using quantitative RT–PCR by comparing results from 36 patients with pancreatic cancer and 30 volunteers. (3) Evaluation of whether plasma miR-18a expression could be used to monitor tumour dynamics by plasma miR-18a assay in patients with pancreatic cancer.

### MiR-18a in primary pancreatic cancer tissues and pancreatic cancer cell lines

**Figure 2:**
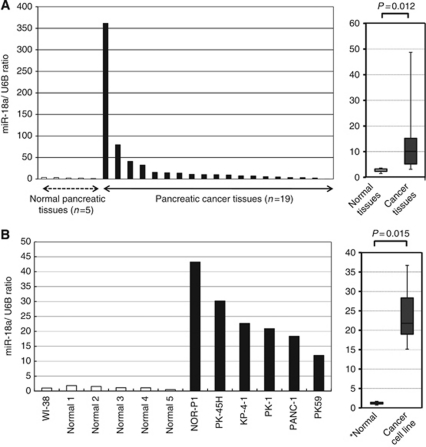
(**A**) The concentrations of miR-18a in pancreatic cancer tissues. The differential expression of miR-18a in pancreatic cancer tissues was compared with that in normal tissues by a waterfall plot (panel **A**). The levels of miR-18a were significantly higher in cancer tissues (*P*=0.012). The upper and lower limits of the boxes and the lines inside the boxes indicate the 75th and 25th percentiles and the median, respectively. The upper and lower horizontal bars denote the 90th and 10th percentiles, respectively. (**B**) Concentrations of miR-18a in pancreatic cancer cell lines. The differential expression of miR-18a in pancreatic cancer cell lines was compared with that in a fibroblast cell line and normal tissues by a waterfall plot (panel **B**). In pancreatic cell lines, levels of miR-18a were significantly higher than those in fibroblasts and normal pancreatic tissues (*P*=0.015). ^*^Normal means normal tissues, including the normal fibroblast cell line WI-38.

To confirm previously reported high miR-18a expression levels in primary pancreatic cancers (Szafranska *et al*, 2007), the expression of miR-18a in 19 pancreatic cancer tissues and in 5 normal pancreatic tissues was determined by quantitative RT–PCR. In addition, the human pancreatic cancer cell lines NOR-P1, PK-45H, KP4-1, PANC-1 and PK-59 and the human fibroblast cell line WI-38 were evaluated by quantitative RT–PCR. Results are shown in Figure 2 after normalisation to the control U6 expression. The expression levels of miR-18a were significantly higher in pancreatic cancer tissues than in normal pancreatic tissues (*P*=0.012) (Figure 2A). Moreover, in pancreatic cell lines, the expression levels of miR-18a were significantly higher than those in the fibroblast cell line and normal pancreatic tissues (*P*=0.015) (Figure 2A). Consequently, the expression of miR-18a was increased in most pancreatic cancer tissues and cell types, but not in normal tissues and fibroblasts.

### Evaluation of miR-18a expression using quantitative RT–PCR in pancreatic cancer patients

Next, we hypothesised that the higher miR-18a expression in primary pancreatic cancer tumours would influence the plasma levels of miR-18a in pancreatic cancer patients. To evaluate the appropriateness of this assay, we first conducted amplification by real-time RT–PCR of a 10-fold serial dilution of the mirVana miRNA reference panel. The linearity of the quantitative RT–PCR was confirmed from concentrations of 1–0.0001 fmol of each synthetic miRNA, such as miR-18a (R2=0.9919) between the logarithm of the amount of input miRNAs and the Ct values (Supplementary Figure S1). Although some investigators have determined quantities of plasma miRNAs by comparing internal control miRNAs (Ng *et al*, 2009; Resnick *et al*, 2009), it remains controversial which miRNAs are suitable as internal control for plasma assays. Therefore, we confirmed a linear correlation between the logarithm of the amount of input synthetic miRNA and the cycle threshold value on real-time PCR, as well as the feasibility of extracting total RNA and amplifying specific miRNA in plasma samples. On the basis of these findings, we used the absolute concentration for measuring the plasma miRNA in this study.

**Figure 3:**
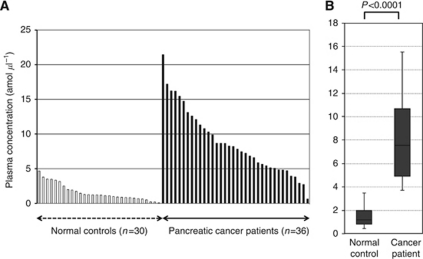
Plasma miR-18a concentration in 36 pancreatic cancer patients and 30 healthy volunteers. Using a real-time RT–PCR assay, circulating miRNA such as miR-18a was detectable in all samples from 36 pancreatic cancer patients and 30 volunteers. (**A**) The differential expression of plasma miR-18a with pancreatic cancer patients was compared with that of normal healthy volunteers by a waterfall plot. Concentrations of miR-18a were significantly higher in the plasma from cancer patients than from volunteers (*P*<0.0001). The upper and lower limits of the boxes and the lines inside the boxes indicate the 75th and 25th percentiles and the median, respectively. (**B**) The upper and lower horizontal bars denote the 90th and 10th percentiles, respectively.

**Figure 4:**
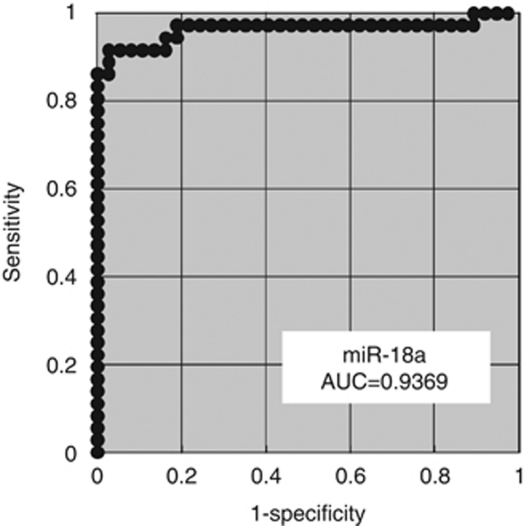
Receiver-operating characteristic (ROC) curve analysis in the miR-18a assay for detecting pancreatic cancer. The ROC analysis showed the greatest AUC of 0.9369 for miR-18a.

Using this assay, circulating miRNA such as miR-18a was detectable in all samples from the 36 pancreatic cancer patients and 30 volunteers. The differential expression of miR-18a in pancreatic cancer patients was compared with that in normal volunteers by a waterfall plot (Figure 3A). Concentrations of miR-18a were significantly higher in the plasma from cancer patients (*P*<0.0001) (Figure 3B). Re-presentation of the data using an ROC plot showed strong separation between the two groups, with an AUC of 0.9369 (Figure 4).

We examined the association of plasma miR-18a concentrations with clinicopathological factors in 36 consecutive pancreatic cancer patients. Table 1 shows the expression in plasma miR-18a levels. We cannot present the correlations between the miR-18a level and clinical factors.

### Evaluation of the use of this assay for monitoring tumour dynamics in patients with pancreatic cancer

**Figure 5:**
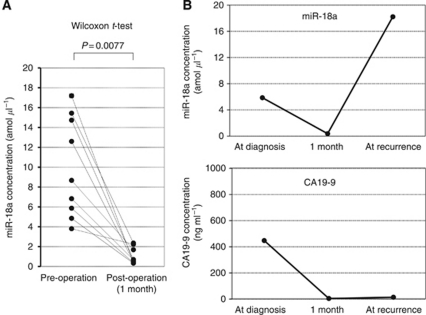
Comparison of plasma miR-18a concentrations between pre- and post-operative samples from pancreatic cancer patients. (**A**) The concentration of miR-18a in the plasma was significantly decreased in post-operative samples compared with the levels in pre-operative samples (*P*=0.0077). (**B**) In one patient, a re-elevation of the plasma miR-18a concentration was found at recurrence after surgery, despite the lack of any elevation in conventional serum tumour markers, such as carbohydrate antigen 19-9 (CA 19-9).

We determined a cutoff value of 6.7 for the plasma miR-18a concentration based on the mean value +2 s.d. in healthy volunteer controls to distinguish high miR-18a concentration patients from low-concentration patients. As a result, 10 patients were categorised into the high-plasma concentration group. We compared the miR-18a expression between plasma and pancreatic cancer tissues in each high-concentration patient. Consequently, in all high-plasma miR-18a concentration patients analysed, miR-18a showed higher expressions in primary pancreatic cancer tissues (100%) than the cutoff value of 4.7 for the tissue miR-18a/U6B ratio based on the mean value+2 s.d. in normal pancreatic tissues (Supplementary Table S1). Moreover, the concentration of miR-18a was analysed in paired pre- and post-operative plasma samples obtained from eight pancreatic cancer patients who underwent curative pancreatectomy, and found to be significantly reduced in the post-operative samples (*P*=0.0077) (Figure 5A). These findings indicated that the level of plasma miR-18a in the plasma might reflect the expression in the tumour. In one patient, a re-elevation of the plasma miR-18a concentration was found at recurrence after surgery, despite the lack of any elevation in conventional serum tumour markers such as CA19-9 (Figure 5B). Furthermore, no correlation was identified between plasma miR-18a levels and CA19-9 (*P*=0.2521) (data not shown). These results clearly showed that the detection of miR-18a in the plasma should provide a new complementary tumour marker for pancreatic cancer.

## Discussion

Numerous genetic and epigenetic changes are involved in tumourigenesis and the progression of various cancers. Several studies have identified tumour-specific alterations to nucleic acids in the plasma/serum of cancer patients, and have demonstrated the potential of circulating nucleic acids as new non-invasive biomarkers in patients with various cancers (Sidransky, 1997; Anker *et al*, 2001; Taback and Hoon, 2004; Chan and Lo, 2007; Diehl *et al*, 2008). In particular, during the last decade, miRNAs have been demonstrated to regulate gene expression by targeting mRNAs for translational repression or cleavage. Consequently, these miRNAs have become known as new factors related to oncogenesis and the progression of various tumours (He *et al*, 2005; Lu *et al*, 2005; Calin and Croce, 2006; He and Hristova, 2008).

MicroRNAs have been proven to contribute to carcinogenesis and may provide new therapeutic strategies as biomarkers and therapeutic targets for cancers. Several studies have shown unique miRNA expression profiles in a number of human tumours, including cancer of the breast, lung, oesophagus, prostate and pancreas, and the differential expression of miRNAs correlates with important histopathological features, such as tumour stage, proliferative capacity and vascular invasion (Lynam-Lennon *et al*, 2009). In particular, studies investigating plasma miRNAs comprise an extremely promising field for clinical application. Tumour-derived miRNA was first described in the plasma by Mitchell *et al* (2008). Plasma miRNAs such as miR-141 could be used to identify prostate cancer patients and show the potential to be new biomarkers. They also exhibited high stability after prolonged incubation at room temperature and/or multiple freezing-thawing processes (Mitchell *et al*, 2008). In addition to this stability, the characteristics of miRNAs such as tissue-specific miRNA signatures and the availability of many copies per cell would indicate potential advantages as biomarkers compared with circulating DNA and mRNA. In fact, accumulating reports also suggest the potential of miRNAs in the early detection of several malignancies, such as prostate cancer (Mitchell *et al*, 2008), lymphoma (Lawrie *et al*, 2008), oral cancer (Wong *et al*, 2008), colorectal cancer (Ng *et al*, 2009), ovarian cancer (Resnick *et al*, 2009), lung cancer (Hu *et al*, 2010), breast cancer (Heneghan *et al*, 2010), gastric cancer (Tsujiura *et al*, 2010) and oesophageal cancer (Komatsu *et al*, 2011).

In pancreatic cancer, it has been reported that alterative expression profiles of miRNAs could distinguish malignant lesions from normal pancreatic tissues and chronic pancreatitis (Roldo *et al*, 2006; Bloomston *et al*, 2007; Szafranska *et al*, 2007). With regard to circulating miRNAs, there have been several reports on the role of circulating miRNAs in the plasma of patients with pancreatic cancer (Wang *et al*, 2009; Ho *et al*, 2010). Wang *et al* (2009) reported that the combined analyses of four different miRNAs, namely miR-21, miR-210, miR-155 and miR-196a, in the plasma could distinguish pancreatic cancer patients from healthy individuals. This report prompted us to detect a more useful plasma miRNA with a clinically satisfactory degree of sensitivity and specificity.

In this study, we selected miR-18a as a new candidate miRNA, which was located in the miR-17–92 cluster and previously reported to present high levels in pancreatic cancers and cell lines (Szafranska *et al*, 2007). The miR-18a miRNA was expressed as part of a cluster of miR-17–92, including miR-17, miR-18a, miR-19a, miR-20a and miR-92 (He *et al*, 2005). This cluster of miRNAs was reported as potential oncogenes in various tumours, with its elevation being caused by genome amplification or by transcriptional activation by MYC (Woods *et al*, 2007).

The amounts of secretory miRNAs are upregulated in the plasma of patients bearing tumours, including B-cell lymphoma, prostate cancer, lung cancer and ovarian cancer. Thus, detection and monitoring of tumours are now becoming possible by the evaluation of tumour-derived secretory miRNAs. Kosaka *et al* (2010) insisted that miRNAs could be incorporated into exosomes and released. In contrast, Arroyo *et al* (2011) recently reported that most miRNAs, including miR-18a, were stable in the plasma by binding the Argonatute2 protein, although some miRNAs were incorporated into exosomes; however, the secretory mechanisms and biological function of extracellular miRNAs remain unclear. At our institute, these issues are also under evaluation.

We hypothesised that if an oncomir such as miR-18a shows strong expression in primary pancreatic cancer tissues, it would influence plasma levels in pancreatic cancer patients. We also hypothesised that the plasma miR-18a concentration would be a potentially useful biomarker in patients with pancreatic cancer. Furthermore, we investigated whether a plasma oncomir such as miR-18a could be released from pancreatic cancers. Comparison between the expression of miR-18a in the plasma and pancreatic cancer tissue samples demonstrated that both samples showed similar tendencies regarding the expression of miR-18a in almost all cases (Supplementary Table S1). However, some patients showed a different pattern of miRNA level; a low plasma miR-18a with high expression in pancreatic cancer tissues. The reasons for the discrepancies in some patients remain to be clarified. One possible explanation for this finding might be the heterogeneity of primary tumours. We also measured circulating miRNAs in paired-plasma obtained before and 1 month after surgical removal of the tumours, to confirm the release of circulating miRNAs. As a result, concentrations of miR-18a were significantly reduced postoperatively in patients with high preoperative plasma miR-18a levels. With regard to the monitoring of cancer, in one representative patient with recurrence, a re-elevation of the plasma miR-18a concentration was found at recurrence after surgery, although there was no elevation of conventional serum tumour markers such as CA19-9. These findings also clearly demonstrated that the plasma concentration of miR-18a reflects tumour dynamics and is available as a new plasma biomarker for monitoring cancer. Although the kinetics and metabolism of plasma miRNAs have not yet been clearly elucidated, this issue is currently under evaluation.

We present here a framework to assess tumour characteristics of pancreatic cancer by a non-invasive plasma miR-18a assay. Although the sample size is quite small, the presence of extremely elevated miR-18a levels in the plasma of pancreatic cancer patients suggests that this may be an apparent benefit in this lethal disease. On the basis of the sensitivity of quantitative RT–PCR, we believe that plasma miR-18a levels should facilitate the earlier diagnosis of pancreatic cancer in the future. However, many issues must be addressed before these findings can be translated into a clinically useful, non-invasive screening strategy for pancreatic cancer patients. These issues are currently under evaluation in a large number of studies, including other promising miRNA candidates in pancreatic cancer patients.

In conclusion, this study clearly demonstrated that plasma miRNAs such as miR-18a provide a useful biomarker for screening pancreatic cancer and monitoring tumour dynamics. The abundance and stability of miRNAs in the plasma further indicate that these blood-based biomarkers have great potential for use to predict the clinical behaviour of individual cancers and to monitor therapeutic responses.

## Figures and Tables

**Table 1:** Pancreatic cancer patient characteristics and plasma miR-18a concentration

**Variables**	**Patients (*n*=36)**	**Plasma miR-18a concentration (amol/*μ*l)^a^**	***P*-value^b^**
*Age (years)*			
Mean (range)	68 (42–84)		
<65	12 (33%)	9.4	0.21
65≦	24 (67%)	8.5	
			
*Sex*			
Male	21 (58%)	9.8	0.11
Female	15 (42%)	7.3	
			
*Tumour size (size)*			
<2	3 (14%)	9.9	0.17
2≦	18 (86%)	10.1	
			
*Histopathology*			
Adenocarcinoma	33 (91%)	8.3	0.06
Adenosquamous	2 (6%)	12.1	
Anaplastic carcinoma	1 (3%)	17.2	
			
*T stage* c			
T1	0 (0%)	—	0.48
T2	2 (7%)	8.0	
T3	22 (76%)	9.6	
T4	5 (17%)	9.1	
			
*N stage* c			
N0	9 (31%)	10.2	0.29
N1	20 (69%)	9.1	
			
*M stage* c			
M0	22 (76%)	9.6	0.48
M1	7 (24%)	8.7	
			
*Stage* c			
I b	1 (3%)	5.7	0.36
II a	7 (24%)	10.5	
II b	13 (45%)	9.0	
III	0 (0%)	—	
IV	8 (28%)	9.7	
			
*Treatment*			
Resectable			
Pancreatectomy	21 (58%)	9.5	0.07
Non-resectable			
Palliative surgery	6 (17%)	7.7	
Far advanced	2 (6%)	13.3	
Recurrence	7 (19%)	6.1	

Abbreviation: miRNA=microRNA.

a Cutoff value of plasma miR-18a concentration in healthy volunteers (mean+2 s.d.): 6.7 amol/*μ*l.

b The Mann–Whitney *U*-test and Kruskal–Wallis *H*-test were performed to compare the plasma miRNA concentration.

c These are according to TMN classification.
