# Supplementary material for: Novel diagnostic value of circulating miR-18a in plasma of patients with pancreatic cancer
Source: Br J Cancer. 2011 Nov 1;105(11):1733–40. doi: 10.1038/bjc.2011.453 (PMC3242609; doi:10.1038/bjc.2011.453)
Supplement: Supplementary Figure S1 and Table S1 [file bjc2011453x1.ppt]

## Slide 1
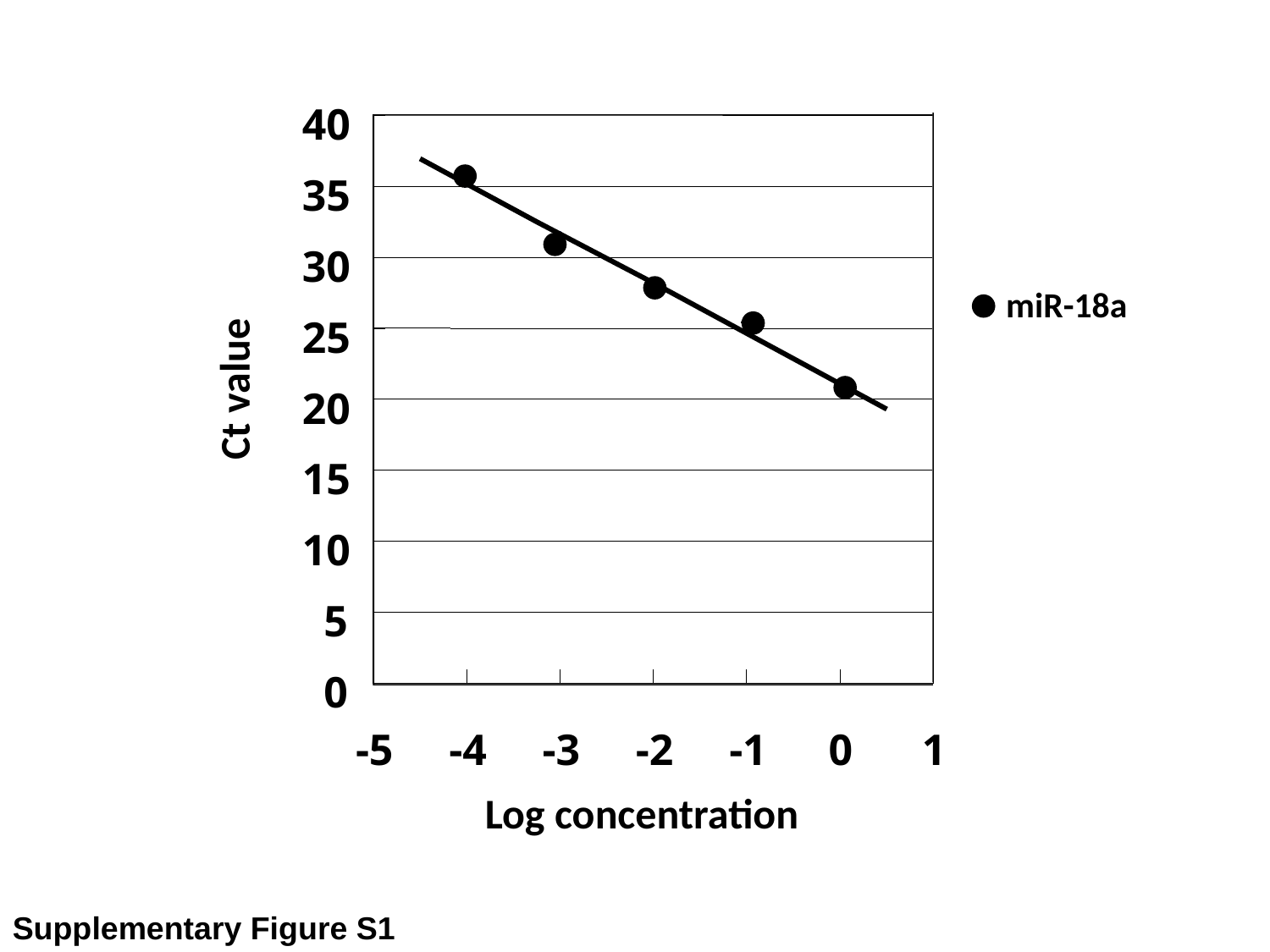

40
35
30
25
20
15
10
5
0
-5
-4
-3
-2
-1
0
1
miR-18a
Ct value
Log concentration
Supplementary Figure S1

## Slide 2
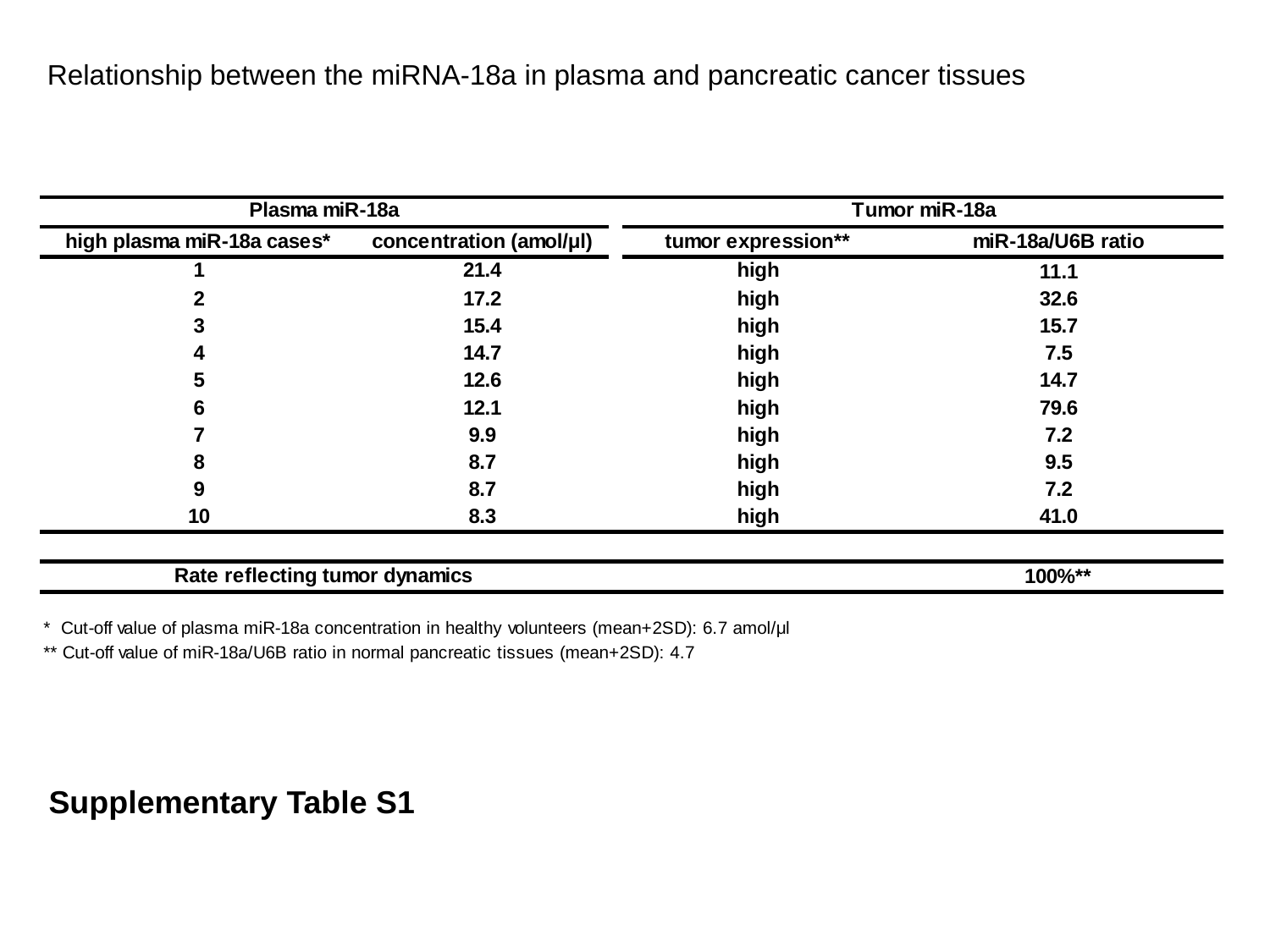

Relationship between the miRNA-18a in plasma and pancreatic cancer tissues
Supplementary Table S1
