# Supplementary material for: Novel diagnostic value of circulating miR-18a in plasma of patients with pancreatic cancer
Source: Br J Cancer. 2011 Nov 1;105(11):1733–40. doi: 10.1038/bjc.2011.453 (PMC3242609; doi:10.1038/bjc.2011.453)
Supplement: Supplementary Figure Legend [file bjc2011453x2.doc]

**Supplementary Figure legends**

**Supplementary Figure S1**

**Standard curve of miR-18a using synthetic microRNAs**

Ten-fold serial dilutions of synthetic microRNA were used to generate the standard curves. Linearity was confirmed within these concentrations, ranging from 1fmol to 0.0001 fmol. (MiR-18a: y = -3.5253x + 21.112(R2 = 0.9919))
